# An Improvement of Robust Biometrics-Based Authentication and Key Agreement Scheme for Multi-Server Environments Using Smart Cards

**DOI:** 10.1371/journal.pone.0145263

**Published:** 2015-12-28

**Authors:** Jongho Moon, Younsung Choi, Jaewook Jung, Dongho Won

**Affiliations:** Department of Computer Engineering, Sungkyunkwan University, Suwon, Gyeonggido 16419, Korea; Nankai University, CHINA

## Abstract

In multi-server environments, user authentication is a very important issue because it provides the authorization that enables users to access their data and services; furthermore, remote user authentication schemes for multi-server environments have solved the problem that has arisen from user’s management of different identities and passwords. For this reason, numerous user authentication schemes that are designed for multi-server environments have been proposed over recent years. In 2015, Lu et al. improved upon Mishra et al.’s scheme, claiming that their remote user authentication scheme is more secure and practical; however, we found that Lu et al.’s scheme is still insecure and incorrect. In this paper, we demonstrate that Lu et al.’s scheme is vulnerable to outsider attack and user impersonation attack, and we propose a new biometrics-based scheme for authentication and key agreement that can be used in multi-server environments; then, we show that our proposed scheme is more secure and supports the required security properties.

## Introduction

Since Lamport [1] proposed the first password-based authentication scheme for insecure communications in 1981, password-based authentication schemes [2–6] have been extensively investigated. The remote user authentication scheme is one of the most convenient authentication schemes for dealing with the transmission of secret data over insecure communication channels, and during the last two decades, many researchers have proposed different remote user authentication schemes.

A problem that occurs with respect to password-based authentication schemes, however, is that a server must maintain a password table for the verification of the legitimacy of a login user; therefore, the server requires additional memory space to store the password table. For this reason, many researchers have proposed a new type of remote user authentication scheme whereby the biological characteristics of persons such as a fingerprint or an iris are used. The main advantageous property of biometrics is uniqueness, leading to the proposal of numerous remote user authentication schemes [7–13] that use biological characteristics. In 2008, Tsai [14] proposed an efficient multi-server authentication scheme using a random number and the one-way hash function; after that, a considerable succession of authenticated key agreement schemes was presented for multi-server environments [15–17]. In 2012, Li et al. [18] proposed a novel authenticated key exchange scheme for multi-server environments; unfortunately, however, Xue et al. [19] found that Li et al.’s scheme did not resist some types of known attacks such as replay, denial of service, forgery, and off-line password guessing. Xue et al. therefore proposed an improved scheme to remedy the weaknesses of Li et al.’s scheme; nevertheless, Lu et al. [20] showed that Xue et al.’s scheme is not only very insecure against impersonation and insider attacks, but that it is also vulnerable to off-line password guessing attack. To overcome the vulnerability of Xue et al.’s scheme, Lu et al. then proposed a slightly modified authentication scheme for multi-server environments. Recently, Chuang et al. [21] presented an efficient, biometrics-based, smart card authentication scheme for a multi-server environment that was previously considered as one that comprises more security properties; however, Mishra et al. [22] found that Chuang et al.’s scheme is vulnerable to a stolen smart card, server spoofing, and impersonation attacks. Mishra et al. also proposed an improved biometrics-based, multi-server authenticated key agreement scheme for which smart cards are used, and they claimed that their scheme satisfied all of the desirable security requirements; unfortunately, Lu et al. [23] showed that Mishra et al.’s scheme did not satisfy key security attributes including replay attack and the incorrect password change phase. Lu et al. then proposed a biometrics-based smart card scheme for authentication and key agreement that can be used in multi-server environments, claiming that their scheme is secure against a variety of known attacks; however, we found that Lu et al.’s scheme is still insecure and is incorrect regarding the login and authentication phase.

In this paper, we concentrate on the security weaknesses of Lu et al.’s biometrics-based authentication scheme. After a careful analysis, we found that their scheme does not effectively resist outsider and impersonation attacks; to resolve these security vulnerabilities, we propose a new biometrics-based scheme for authentication and key agreement that can be used in a multi-server environment. In addition, we demonstrate that the proposed scheme provides a strong authentication defense against a number of attacks including the attacks of the original scheme. Lastly, we compare the performance and functionality of the proposed scheme with other related schemes.

The rest of the paper is organized as follows: In section 2 and section 3, we review and analyze, respectively, Lu et al.’s scheme; in Section 4, we propose an improved authentication scheme for multi-server environments; in section 5, we present a security analysis of our scheme; section 6 shows security and performance analyses whereby our scheme is compared with previous schemes; and, our conclusion is presented in section 7.

## Review of Lu et al.’s scheme

In this section, we will review Lu et al.’s biometrics-based scheme for authentication and key agreement that can be used in a multi-server environment. The following three participants are involved: the user *U*_*i*_, the server *S*_*j*_, and the registration center *RC*. The *RC* chooses a secret key *PSK* and a secret number *x* and shares them with *S*_*j*_ over a secure channel. The scheme consists of the registration, login and authentication, and password updating. For convenience, some of the notations that are used in Lu et al.’s scheme are described in Table 1.

**Table 1:** Notations used in Lu et al.’s scheme.

*U*_*i*_, *S*_*j*_	User and a server
*RC*	The registration center
*ID*_*i*_, *SID*_*j*_	Identity of *U*_*i*_ and *S*_*j*_
*PW*_*i*_, *BIO*_*i*_	Password and a biometrics of *U*_*i*_
*x*, *y*	Secret number selected by the *RC* and *U*_*i*_
*PSK*	Secure key shared by the *RC* and *S*_*j*_
*T*	Timestamp
*h*(⋅)	One-way hash function
*H*(⋅)	Biohash function
⊕, ∥	Exclusive-or operation and concatenation operation

### Registration

1. *U*_*i*_ enters his/her biometrics *BIO*_*i*_, identity *ID*_*i*_ and password *PW*_*i*_; then, *U*_*i*_ sends {*ID*_*i*_, *h*(*PW*_*i*_ ∥ *H*(*BIO*_*i*_))} to the *RC*.
2. After receiving the message from *U*_*i*_, the *RC* computes *X*_*i*_ = *h*(*ID*_*i*_ ∥ *x*), *V*_*i*_ = *h*(*ID*_*i*_ ∥ *h*(*PW*_*i*_ ∥ *H*(*BIO*_*i*_))); then, the *RC* stores {*X*_*i*_, *V*_*i*_, *h*(*PSK*)} onto a smart card and submits them to *U*_*i*_.
3. *U*_*i*_ computes *Y*_*i*_ = *h*(*PSK*) ⊕ *y*, and replaces *h*(*PSK*) with *Y*_*i*_, lastly, the smart card stores the values of {*X*_*i*_, *Y*_*i*_, *V*_*i*_, *h*(⋅)}.

### Login and authentication

1. *U*_*i*_ inserts his/her smart card into the device and enters his/her identity *ID*_*i*_, password *PW*_*i*_ and biometrics *BIO*_*i*_; then, the smart card validates whether $V_{i}^{\prime }=h\left(ID_{i}∥h\left(PW_{i}∥H\left(BIO_{i}\right)\right)\right)$ is equal to the stored *V*_*i*_; if validation occurs, the smart card generates a random number *n*_1_ and computes *K* = *h*((*Y*_*i*_ ⊕ *y*) ∥ *SID*_*j*_), *M*_1_ = *K* ⊕ *ID*_*i*_, *M*_2_ = *n*_1_ ⊕ *K*, *M*_3_ = *h*(*PW*_*i*_ ∥ *H*(*BIO*_*i*_)) ⊕ *K*, and *Z*_*i*_ = *h*(*X*_*i*_ ∥ *n*_1_ ∥ *h*(*PW*_*i*_ ∥ *H*(*BIO*_*i*_)) ∥ *T*_1_). Lastly, *U*_*i*_ sends {*Z*_*i*_,*M*_1_,*M*_2_,*M*_3_,*T*_1_} to *S*_*j*_ over a public channel, where *T*_1_ is the current timestamp.
2. After receiving the message from *U*_*i*_, *S*_*j*_ first checks whether *T*_*c*_ − *T*_1_ ≤ △*T* and then computes *K* = *h*(*SID*_*j*_ ∥ *h*(*PSK*)) by using a secure pre-shared key *PSK*; then *S*_*j*_ retrieves *ID*_*i*_ = *M*_1_ ⊕ *K*, *n*_1_ = *M*_2_ ⊕ *K*, *h*(*PW*_*i*_ ∥ *H*(*BIO*_*i*_)) = *M*_3_ ⊕ *K*. *S*_*j*_ subsequently computes *X*_*i*_ = *h*(*ID*_*i*_ ∥ *x*) and verifies whether $h\left(X_{i}∥n_{1}∥h\left(PW_{i}∥H\left(BIO_{i}\right)\right)∥T_{1}\right)\overset{?}{=}Z_{i}$; if it holds, *S*_*j*_ generates a random number *n*_2_ and computes *SK*_*ji*_ = *h*(*n*_1_ ∥ *n*_2_ ∥ *K* ∥ *X*_*i*_), *M*_4_ = *n*_2_ ⊕ *h*(*n*_1_ ∥ *h*(*PW*_*i*_ ∥ *H*(*BIO*_*i*_)) ∥ *X*_*i*_), and *M*_5_ = *h*(*ID*_*i*_ ∥ *n*_1_ ∥ *n*_2_ ∥ *K* ∥ *T*_2_). Then, *S*_*j*_ sends back the authentication message {*M*_4_,*M*_5_,*T*_2_} to *U*_*i*_, where *T*_2_ is the current timestamp.
3. Upon checking the freshness of *T*_2_, *U*_*i*_ first computes *n*_2_ = *M*_4_ ⊕ *h*(*n*_1_ ∥ *h*(*PW*_*i*_ ∥ *H*(*BIO*_*i*_)) ∥ *X*_*i*_) and then verifies whether *h*(*ID*_*i*_ ∥ *n*_1_ ∥ *n*_2_ ∥ *K* ∥ *T*_2_) is equal to the received *M*_5_; if they are equal, *U*_*i*_ computes the common session key *SK*_*ij*_ = *h*(*n*_1_ ∥ *n*_2_ ∥ *K* ∥ *X*_*i*_) and sends {*M*_6_ = *h*(*SK*_*ij*_ ∥ *ID*_*i*_ ∥ *n*_2_ ∥ *T*_3_), *T*_3_} to *S*_*j*_, where *T*_3_ is the current timestamp.
4. *S*_*j*_ verifies the freshness of *T*_3_ and the correctness of *M*_6_ by using *SK*_*ji*_, and if they do not hold, *S*_*j*_ stops the execution; otherwise, *S*_*j*_ confirms the common session key *SK*_*ji*_ with *U*_*i*_.

### Password updating

*U*_*i*_ first inputs his/her smart card into the device and provides his/her identity *ID*_*i*_, password *PW*_*i*_ and biometrics *BIO*_*i*_. The smart card then validates whether $V_{i}^{\prime }=h\left(ID_{i}∥h\left(PW_{i}∥H\left(BIO_{i}\right)\right)\right)$ is equal to the stored *V*_*i*_; if they are equal, *U*_*i*_ keys in the new password *PW*_*i*(*new*)_, but otherwise the smart card refuses the request. Lastly, the smart card computes *V*_*i*(*new*)_ = *h*(*ID*_*i*_ ∥ *h*(*PW*_*i*(*new*)_ ∥ *H*(*BIO*_*i*_))) and replaces *V*_*i*_ by *V*_*i*(*new*)_.

## Security analysis of Lu et al.’s scheme

According to [24, 25], in the basic adversary model, a probabilistic polynomial-time (PPT) adversary A can have a full control over all communication messages. The adversary A then can read, modify or delete all communication messages transmitted between a user and the server. Furthermore, power analysis attacks [26] can extract all of the information from the smart card by using the side channel attack. Lu et al. claimed that their scheme could resist a session-key attack; however, we demonstrated that their scheme is still insecure against a session key attack. We also found that their scheme is unable to provide protection against outsider and user impersonation attacks, and it cannot support user anonymity; furthermore, a number of the phases of Lu et al.’s scheme are not correct and we point out the details of these problems in the following subsections.

### Incorrect login phase

During the login phase, the user *U*_*i*_ inserts his/her smart card into the card reader, inputs his/her identity *ID*_*i*_, password *PW*_*i*_, and then imprints his/her biometrics *BIO*_*i*_ at the sensor. The smart card then validates whether $V_{i}^{\prime }=h\left(ID_{i}∥h\left(PW_{i}∥H\left(BIO_{i}\right)\right)\right)$ is equal to the stored *V*_*i*_; if it holds, the smart card should compute *K* = *h*((*Y*_*i*_ ⊕ *y*) ∥ *SID*_*j*_), but this is actually impossible because the secret key *y* does not exist in the smart card. Lu et al. claimed that even if an adversary A has gathered the information {*X*_*i*_,*Y*_*i*_,*V*_*i*_,*h*(⋅)} that is stored in *U*_*i*_’s smart card, A cannot figure out the login request message {*Z*_*i*_,*M*_1_,*M*_2_,*M*_3_,*T*_1_} without the secret key *y*; therefore, we assumed that the secret key *y* is entered by user *U*_*i*_ during the login process.

### Incorrect authentication phase

During the authentication phase, the server *S*_*j*_ computes *K* = *h*(*SID*_*j*_ ∥ *h*(*PSK*)) by using a secure pre-shared key *PSK*; however, the value *K* = *h*(*SID*_*j*_ ∥ *h*(*PSK*)) cannot be made equal to *K* = *h*((*Y*_*i*_ ⊕ *y*) ∥ *SID*_*j*_) = *h*(*h*(*PSK*)∥*SID*_*j*_) by computing *U*_*i*_. We therefore assumed that server *S*_*j*_ computes *K* = *h*(*h*(*PSK*) ∥ *SID*_*j*_)).

### Outsider Attack

During the registration phase, the *RC* stores {*X*_*i*_,*V*_*i*_,*h*(*PSK*)} onto a smart card and submits them to *U*_*i*_. After receiving the smart card, *U*_*i*_ computes *Y*_*i*_ = *h*(*PSK*) ⊕ *y*, and replaces *h*(*PSK*) with *Y*_*i*_. Let A who is in possession of the smart card extracted information $\left\{X_{A},V_{A},h\left(PSK\right)\right\}$, be an active adversary of the legal user; then, A can easily compute *K* = *h*(*h*(*PSK*)||*SID*_*j*_) that is the same for each legal user that belongs in the server *S*_*j*_. Furthermore, if A intercepts his/her own login request message $\left\{Z_{A},M_{1},M_{2},M_{3},T_{1}\right\}$, then A can also compute $K=M_{3}⊕h\left(PW_{A}∥H\left(BIO_{A}\right)\right)$.

### Violation of the Session Key Security

Suppose an outsider adversary A intercepts the communication between *U*_*i*_ and *S*_*j*_ and steals the smart card of *U*_*i*_; then, he/she can obtain all of the messages {*Z*_*i*_,*M*_1_,*M*_2_,*M*_3_,*M*_4_,*M*_5_,*M*_6_,*T*_1_,*T*_2_,*T*_3_} and extract the information {*X*_*i*_,*Y*_*i*_,*V*_*i*_,*h*(⋅)}, thereby easily obtaining the session key that is transmitted between *U*_*i*_ and *S*_*j*_. The details are described as follows.

1. A computes *n*_1_ = *M*_2_ ⊕ *K*, *ID*_*i*_ = *K* ⊕ *M*_1_, and *h*(*PW*_*i*_ ∥ *H*(*BIO*_*i*_)) = *M*_3_ ⊕ *K*.
2. Then, A can compute *n*_2_ = *M*_4_ ⊕ *h*(*n*_1_ ∥ *h*(*PW*_*i*_ ∥ *H*(*BIO*_*i*_)) ∥ *X*_*i*_); therefore, A can obtain the session key *SK*_*ij*_ = *h*(*n*_1_ ∥ *n*_2_ ∥ *K* ∥ *X*_*i*_).

### User Impersonation Attack

As described in this subsection, A can also impersonate as a legal user to cheat *S*_*j*_ when he/she knows the value of *K*. The details are described as follows.

1. A generates a random number $n_{1}^{\prime }$ and computes *M*_1_ = *K* ⊕ *ID*_*i*_, $M_{2}=n_{1}^{\prime }⊕K$, *M*_3_ = *K* ⊕ *h*(*PW*_*i*_ ∥ *H*(*BIO*_*i*_)) and $Z_{i}=h\left(X_{i}∥n_{1}^{\prime }∥h\left(PW_{i}∥H\left(BIO_{i}\right)\right)∥T_{1}^{\prime }\right)$; then, A sends the login request message $\left\{Z_{i},M_{1},M_{2},M_{3},T_{1}^{\prime }\right\}$ to server *S*_*j*_, where $T_{1}^{\prime }$ is the current timestamp.
2. After receiving the login request message from A who pretends to be *U*_*i*_, the message can successfully pass *S*_*j*_’s verification and *S*_*j*_ performs the subsequent scheme normally. Lastly, *S*_*j*_ sends the authenticated message $\left\{M_{4},M_{5},T_{2}^{\prime }\right\}$ to A, where $n_{2}^{\prime }$ and $T_{2}^{\prime }$ are the random number and the current timestamp on the server side, respectively.
3. Upon receiving the login response message from *S*_*j*_, A computes $n_{2}^{\prime }=M_{4}⊕h(n_{1}^{\prime }||h(PW_{i}||H\left(BIO_{i}\right))||X_{i})$, $SK_{ij}=h\left(n_{1}^{\prime }∥n_{2}^{\prime }∥K∥X_{i}\right)$, and $M_{6}=h\left(SK_{ij}∥ID_{i}∥n_{2}^{\prime }∥T_{3}^{\prime }\right)$, and sends the message $\left\{M_{6},T_{3}^{\prime }\right\}$ to *S*_*j*_, where $T_{3}^{\prime }$ is the current timestamp.
4. Upon receiving the message from A, *S*_*j*_ continues to proceed with the scheme without detection. Lastly, A and *S*_*j*_ “successfully” agree on the session key *SK*_*ij*_, but unfortunately *S*_*j*_ mistakenly believes that he/she is communicating with the legitimate, genuine *U*_*i*_.

### User is not anonymous

Lu et al. claimed that *U*_*i*_’s identity *ID*_*i*_ is well protected by the shared parameter *K* that is used as a substitute for the actual parameters. Additionally, an unauthorized server cannot obtain *ID*_*i*_ without knowing *K*, since *K* is protected by a secret key *PSK* that is only known by the authorized server and is not exposed on the open channel. We found, however, that if the outsider adversary A can obtain *h*(*PSK*), then he/she can compute *K* = *h*(*h*(*PSK*) ∥ *SID*_*j*_); furthermore, A can also compute $K=M_{3}⊕h\left(PW_{A}∥H\left(BIO_{A}\right)\right)$ without *h*(*PSK*), meaning that A can compute *ID*_*i*_ = *M*_1_ ⊕ *K*. We therefore concluded that Lu et al.’s scheme cannot provide user anonymity.

## Our proposed scheme

**Fig 1:**
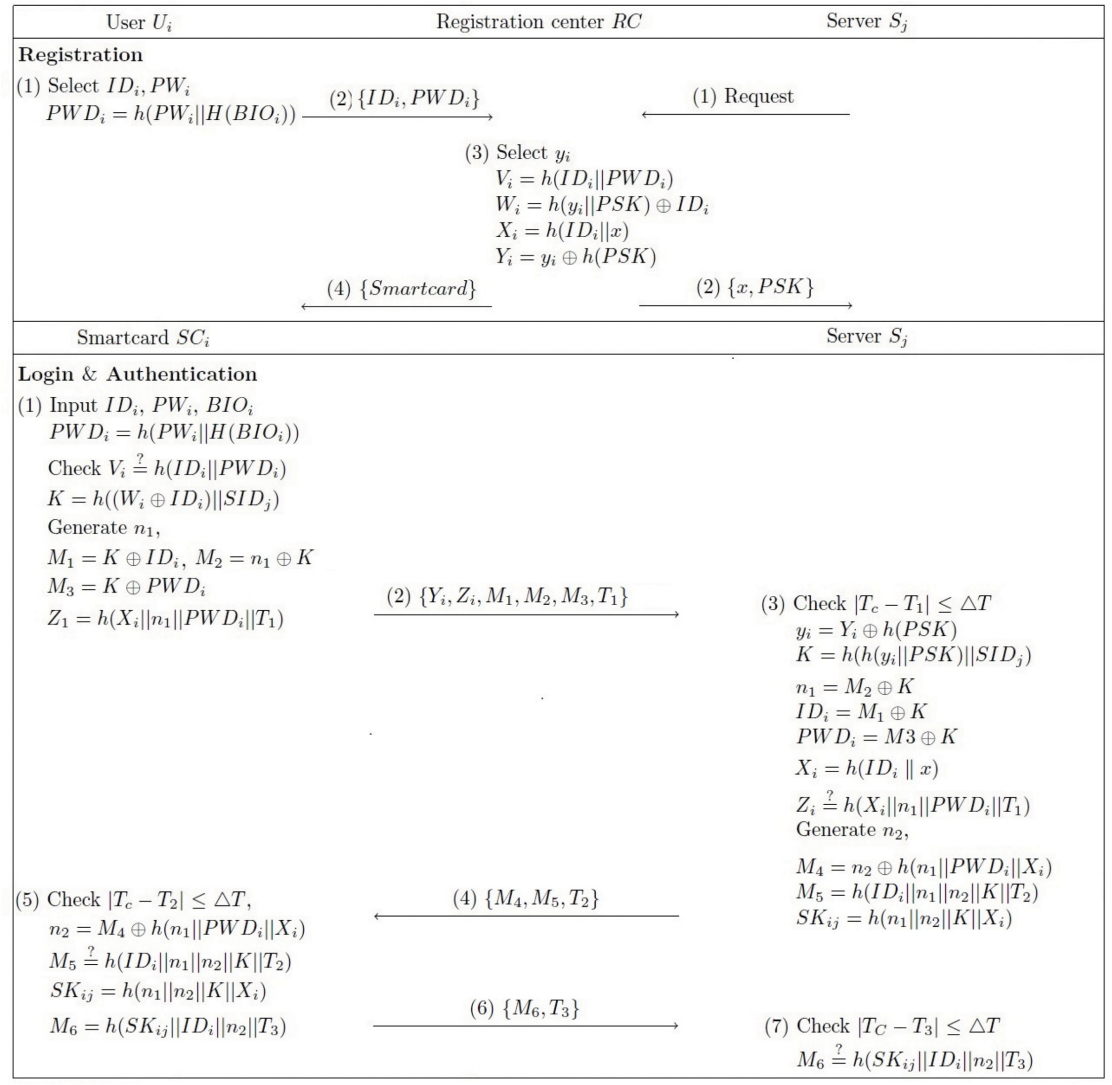
Our proposed authentication and key agreement protocol for multi-server environments.

In this section, we will propose a new biometrics-based password authentication scheme for multi-server environments. In our scheme, there are also three participants, as follows: the user *U*_*i*_, the server *S*_*j*_, and the registration center *RC*. The *RC* chooses a secret key *PSK* and a secret number *x*, and then shares them with *S*_*j*_ over a secure channel. Our proposed scheme consists of the following four phases as shown in Fig 1: registration, login, authentication, and password changing. For convenience, some of the notations that are used in our proposed scheme are described in Table 2.

**Table 2:** Notations used in our proposed scheme.

*U*_*i*_	The *i* ^*th*^ user
*S*_*j*_	The *j* ^*th*^ server
*SC*_*i*_	The smart card of the *i* ^*th*^ user
*RC*	The registration center
*ID*_*i*_	Identity of the *i* ^*th*^ user
*SID*_*j*_	Identity of the *j* ^*th*^ server
*PW*_*i*_	Password of the *i* ^*th*^ user
*BIO*_*i*_	Biometrics of the *i* ^*th*^ user
*x*	A secret number selected by *RC*
*y*_*i*_	A random number unique to user selected by *RC*
*PSK*	Secure key pre-shared by *RC* and *S*_*j*_
*T*	A timestamp
*h*(⋅)	A one-way hash function
*H*(⋅)	Biohash function
⊕, ∥	Exclusive-or operation and concatenation operation

### Registration phase

1. *U*_*i*_ inputs his/her biometrics *BIO*_*i*_ and selects an identity *ID*_*i*_ and a password *PW*_*i*_. Then, *U*_*i*_ computes *PWD*_*i*_ = *h*(*PW*_*i*_ ∥ *H*(*BIO*_*i*_)) and sends {*ID*_*i*_, *PWD*_*i*_} to the *RC*.
2. After receiving the registration request message from *U*_*i*_, the *RC* generates a random number *y*_*i*_ that is unique to *U*_*i*_. Then, the *RC* computes *V*_*i*_ = *h*(*ID*_*i*_ ∥ *PWD*_*i*_), *W*_*i*_ = *h*(*y*_*i*_ ∥ *PSK*) ⊕ *ID*_*i*_, *X*_*i*_ = *h*(*ID*_*i*_ ∥ *x*), and *Y*_*i*_ = *y*_*i*_ ⊕ *h*(*PSK*), followed by the storage of {*V*_*i*_,*W*_*i*_,*X*_*i*_,*Y*_*i*_,*h*(⋅),*H*(⋅)} by the *RC* onto a smart card and the submission of them to *U*_*i*_.
3. The *RC* sends the smart card *SC*_*i*_ to *U*_*i*_ over a secure channel and the registration phase is therefore complete.

### Login phase

1. *U*_*i*_ inserts his/her smart card into the card reader and enters identity *ID*_*i*_, password *PW*_*i*_ and imprints biometrics *BIO*_*i*_; then, the smart card *SC*_*i*_ computes *PWD*_*i*_ = *h*(*PW*_*i*_ ∥ *H*(*BIO*_*i*_)) to validate whether $V_{i}^{\prime }=h\left(ID_{i}∥PWD_{i}\right)$ is equal to the stored *V*_*i*_. If it holds, the smart card generates a random number *n*_1_ and computes *K* = *h*((*W*_*i*_ ⊕ *ID*_*i*_) ∥ *SID*_*j*_), *M*_1_ = *K* ⊕ *ID*_*i*_, *M*_2_ = *n*_1_ ⊕ *K*, *M*_3_ = *PWD*_*i*_ ⊕ *K*, and *Z*_*i*_ = *h*(*X*_*i*_ ∥ *n*_1_ ∥ *PWD*_*i*_ ∥ *T*_1_).
2. *U*_*i*_ then sends {*Y*_*i*_,*Z*_*i*_,*M*_1_,*M*_2_,*M*_3_,*T*_1_} to *S*_*j*_ over a public channel, where *T*_1_ is the current timestamp.

### Authentication phase

1. After receiving the login request message from *U*_*i*_, *S*_*j*_ first checks whether *T*_*c*_ − *T*_1_ ≤ △*T* so that it can then compute *y*_*i*_ = *Y*_*i*_ ⊕ *h*(*PSK*) by using a secure pre-shared key *PSK*; then, *S*_*j*_ computes *K* = *h*(*h*(*y*_*i*_ ∥ *PSK*) ∥ *SID*_*j*_), *ID*_*i*_ = *M*_1_ ⊕ *K*, *n*_1_ = *M*_2_ ⊕ *K*, and *PWD*_*i*_ = *M*_3_ ⊕ *K*. Next, *S*_*j*_ computes *X*_*i*_ = *h*(*ID*_*i*_ ∥ *x*) and verifies whether $h\left(X_{i}∥n_{1}∥PWD_{i}∥T_{1}\right)\overset{?}{=}Z_{i}$. If it holds, *S*_*j*_ generates a random number *n*_2_ and computes *SK*_*ji*_ = *h*(*n*_1_ ∥ *n*_2_ ∥ *K* ∥ *X*_*i*_), *M*_4_ = *n*_2_ ⊕ *h*(*n*_1_ ∥ *PWD*_*i*_ ∥ *X*_*i*_), and *M*_5_ = *h*(*ID*_*i*_ ∥ *n*_1_ ∥ *n*_2_ ∥ *K* ∥ *T*_2_). Then, *S*_*j*_ sends the login response message {*M*_4_,*M*_5_,*T*_2_} to *U*_*i*_ where *T*_2_ is the current timestamp.
2. Upon checking the freshness of *T*_2_, *U*_*i*_ first computes *n*_2_ = *M*_4_ ⊕ *h*(*n*_1_ ∥ *PWD*_*i*_ ∥ *X*_*i*_) and then verifies whether *h*(*ID*_*i*_ ∥ *n*_1_ ∥ *n*_2_ ∥ *K* ∥ *T*_2_) is equal to the received *M*_5_. If they are equal, *U*_*i*_ computes the common session key *SK*_*ij*_ = *h*(*n*_1_ ∥ *n*_2_ ∥ *K* ∥ *X*_*i*_) and sends {*M*_6_ = *h*(*SK*_*ij*_ ∥ *ID*_*i*_ ∥ *n*_2_ ∥ *T*_3_), *T*_3_} to *S*_*j*_, where *T*_3_ is the current timestamp.
3. *S*_*j*_ verifies the freshness *T*_3_ and the correctness of *M*_6_ by using *SK*_*ji*_; if they hold, *S*_*j*_ confirms the common session key *SK*_*ji*_ with *U*_*i*_, but otherwise, *S*_*j*_ terminates this session.

### Password updating

The password change is done locally without the involvement of the *RC*. If *U*_*i*_ wants to change his/her password, he/she first inserts his/her smart card into a card reader and provides his/her identity *ID*_*i*_, password *PW*_*i*_ and biometrics *BIO*_*i*_. The smart card *SC*_*i*_ then computes *PWD*_*i*_ = *h*(*PW*_*i*_ ∥ *H*(*BIO*_*i*_)) to validate whether $V_{i}^{\prime }=h\left(ID_{i}∥PWD_{i}\right)$ is equal to the stored *V*_*i*_. If they are equal, *SC*_*i*_ accepts *U*_*i*_ to enter a new password *PW*_*i*(*new*)_, but otherwise, the smart card rejects the password changing request. Lastly, *SC*_*i*_ computes *PWD*_*i*(*new*)_ = *h*(*PW*_*i*(*new*)_ ∥ *H*(*BIO*_*i*_)), and *V*_*i*(*new*)_ = *h*(*ID*_*i*_ ∥ *PWD*_*i*(*new*)_), and replaces *V*_*i*_ with *V*_*i*(*new*)_.

## Security analysis of our proposed scheme

In this section, we demonstrate that our scheme, which retains the merits of Lu et al.’s scheme, can withstand several types of possible attacks, and we also show that our scheme supports several security properties. The security analysis of our proposed scheme was conducted under the following four assumptions:

1. An adversary A can be either a user or a server. A registered user as well as a registered server can act as an adversary.
2. An adversary A can eavesdrop on every communication across public channels. He/she can capture any message that is exchanged between a user and a server.
3. An adversary A has the ability to alter, delete, or reroute a captured message.
4. Information can be extracted from the a smart card by examining the power consumption of the card.

### Verifying the authentication scheme with BAN logic

Burrows-Abadi-Needham(BAN) logic [27] is a set of rules for the definition and analysis of information exchange protocols. Concretely, BAN logic helps its users to decide whether exchanged information is trustworthy, whether it is secured against eavesdropping, or both. In this subsection, we use BAN logic to prove that a shared session key between a user and a server can be correctly generated during the authentication process. Some of the notations and logical postulates [28] that are used in the BAN logic are described in Table 3.

**Table 3:** Notations used in BAN Logic.

P|≡X	The principal P believes the statement X.
#(X)	The formula X is fresh.
P⇒X	The principal P has jurisdiction over the statement X.
$P\overset{K}{↔}Q$	The principals P and Q may use the shared key K.
P◃X	The principal P sees the statement X.
P|∼X	The principal P once said the statement X.
${\left\{X\right\}}_{K}$	The formula X encrypted under the key K.
${\left(X\right)}_{K}$	The formula X hashed under the key K.
${\langle X\rangle }_{Y}$	The formula X combined with the key Y.
$P\overset{X}{\Leftrightarrow }Q$	The formula X is a secret known only to *P* and *Q*.

1. BAN logical postulates
  - Message-meaning rule: $\frac{P|\equiv P\overset{K}{↔}Q,P◃{\left\{X\right\}}_{K}}{P|\equiv Q|∼X}$: If principal P believes that he/she shares the secret key K with Q, and P sees the statement X encrypted under K. Then P believes that Q once said X.
  - Nonce-verification rule: $\frac{P|\equiv \#(X),P|\equiv Q|∼X}{P|\equiv Q|\equiv X}$: If principal P believes that X is fresh and P believes that Q once said X, then P believes that Q believes X.
  - The belief rule: $\frac{P|\equiv X,P|\equiv Y}{P|\equiv (X,Y)}$: If principle P believes X and Y, then P believes (X,Y).
  - Freshness-conjuncatenation rule: $\frac{P|\equiv \#(X)}{P|\equiv \#(X,Y)}$: If principle P believes that X is fresh, then P believes (X,Y) is fresh.
  - Jurisdiction rule: $\frac{P|\equiv Q|\Rightarrow X,P|\equiv Q|\equiv X}{P|\equiv X}$: If principle P believes that Q has jurisdiction over X and P believes that Q believes X, then P believes X.
2. Idealized scheme
*U_i_*: 〈*y*_*i*_〉_*h*(*PSK*)_, 〈*n*_1_,*ID*_*i*_,*PWD*_*i*_〉_*K*_, (*n*_1_,*X*_*i*_,*T*_1_)_*PWD*_*i*__, ${\left(n_{2},U_{i}\overset{SK_{ij}}{↔S_{j}},T_{3}\right)}_{ID_{i}}$
*S_j_*: 〈*n*_1_,*X*_*i*_,*PWD*_*i*_〉_*n*_2__, (*ID*_*i*_,*n*_1_,*n*_2_,*T*_2_)_*K*_
3. Establishment of security goals
  *g*_1_ $S_{j}\left|\equiv U_{i}\right|\equiv U_{i}\overset{SK_{ij}}{↔}S_{j}$
  *g*_2_ $S_{j}|\equiv U_{i}\overset{SK_{ij}}{↔}S_{j}$
  *g*_3_ $U_{i}\left|\equiv S_{j}\right|\equiv U_{i}\overset{SK_{ji}}{↔}S_{j}$
  *g*_4_ $U_{i}\left|\equiv U_{i}\right|\overset{SK_{ji}}{↔}S_{j}$
4. Initiative premises
  *p*_1_ *U*_*i*_| ≡#*n*_1_, *p*_2_. *U*_*i*_| ≡*S*_*j*_ ⇒ #*n*_2_, *p*_3_. *S*_*j*_| ≡#*n*_1_, *p*_4_. *S*_*j*_| ≡#*n*_2_,
  *p*_5_ $S_{j}|\equiv U_{i}\overset{K}{↔}S_{j}$, *p*_6_. $U_{i}|\equiv U_{i}\overset{K}{↔}S_{j}$, *p*_7_. *U*_*i*_| ≡*ID*_*i*_,
  *p*_8_ *S*_*j*_| ≡*U*_*i*_ ⇒ *PWD*_*i*_, *p*_9_. *S*_*j*_| ≡*U*_*i*_ ⇒ *ID*_*i*_, *p*_10_. *U*_*i*_| ≡*S*_*j*_ ⇒ *X*_*i*_,
  *p*_11_ $S_{j}|\equiv U_{i}\Rightarrow U_{i}\overset{SK_{ij}}{↔}S_{j}$, *p*_12_. $U_{i}|\equiv S_{j}\Rightarrow U_{i}\overset{SK_{ij}}{↔}S_{j}$
5. Our proposed scheme analysis
  *a*_1_ By *p*_5_, *S*_*j*_ ⊲ 〈*y*_*i*_〉_*h*(*PSK*)_, and *S*_*j*_ ⊲ 〈*n*_*i*_,*ID*_*i*_,*PWD*_*i*_〉_*K*_, we apply the message-meaning rule to drive: *S*_*j*_| ≡*U*_*i*_| ∼(*n*_1_,*ID*_*i*_,*PWD*_*i*_)
  *a*_2_ By *a*_1_ and *p*_3_, we apply the fresh conjuncatenation rule and the nonce-verification rule to derive: *S*_*j*_|≡*U*_*i*_|≡(*n*_1_,*ID*_*i*_,*PWD*_*i*_)
  *a*_3_ By *a*_2_, *p*_3_ and *p*_8_, we apply the belief rule and the jurisdiction rule to derive: *S*_*j*_| ≡*ID*_*i*_
  *a*_4_ By *a*_3_ and $S_{j}◃{\left(n_{2},U_{i}\overset{SK_{ij}}{↔}S_{j},T_{3}\right)}_{ID_{i}}$, we apply the message-meaning rule to derive: $S_{j}\left|\equiv U_{i}\right|∼\left(n_{2},U_{i}\overset{SK_{ij}}{↔}S_{j},T_{3}\right)$
  *a*_5_ By *p*_4_ and *a*_4_, we apply the fresh conjuncatenation rule and the nonce-verification rule to drive: $S_{j}\left|\equiv U_{i}\right|\equiv \left(n_{2},U_{i}\overset{SK_{ij}}{↔}S_{j},T_{3}\right)$
  *g*_1_ By *a*_5_, we apply the belief rule to derive: $S_{j}\left|\equiv U_{i}\right|\equiv U_{i}\overset{SK_{ij}}{↔}S_{j}$
  *g*_2_ By *g*_1_ and *p*_1_, we apply the jurisdiction rule to derive: $S_{j}|\equiv U_{i}\overset{SK_{ij}}{↔}S_{j}$
  *a*_6_ By *p*_6_ and *U*_*i*_ ⊲ (*ID*_*i*_,*n*_1_,*n*_2_,*T*_2_)_*K*_, we apply the message-meaning rule to derive: *U*_*i*_| ≡*S*_*j*_|∼(*ID*_*i*_,*n*_1_,*n*_2_,*T*_2_)
  *a*_7_ By *p*_2_ and *a*_6_, we apply the fresh conjuncatenation rule and the nonce-verification rule to derive: *U*_*i*_| ≡*S*_*j*_| ≡(*ID*_*i*_,*n*_1_,*n*_2_,*T*_2_)
  *a*_8_ By *a*_7_, we apply the belief rule to derive: *U*_*i*_| ≡*S*_*j*_| ≡*n*_2_
  *a*_9_ By *p*_2_ and *a*_8_, we apply the jurisdiction rule to derive: *U*_*i*_| ≡*n*_2_
  *a*_10_ By *a*_9_ and *U*_*i*_ ⊲ 〈*n*_1_,*X*_*i*_,*PWD*_*i*_〉_*n*_2__, we apply the message-meaning rule to derive: *U*_*i*_| ≡*S*_*j*_|∼(*n*_1_,*X*_*i*_,*PWD*_*i*_)
  *a*_11_ By *a*_10_ and *p*_1_, we apply the fresh conjuncatenation rule and the nonce-verification rule to derive: *U*_*i*_| ≡*S*_*j*_| ≡(*n*_1_,*X*_1_,*PWD*_*i*_)
  *g*_3_ By *p*_1_, *p*_3_, *p*_4_, *p*_6_, *a*_11_ and *SK*_*ij*_ = *h*(*n*_1_ ∥ *n*_2_ ∥ *K* ∥ *X*_*i*_), we apply the fresh conjuncatenation rule and the nonce-verification rule to derive: $U_{i}\left|\equiv S_{j}\right|\equiv U_{i}\overset{SK_{ij}}{↔}S_{j}$
  *g*_4_ By *g*_3_ and *p*_12_, we apply the jurisdiction rule to derive: $U_{i}|\equiv U_{i}\overset{SK_{ij}}{↔}S_{j}$

### Informal security analysis

In this subsection, we verify whether our proposed scheme is secure against a variety of known attacks.

#### Anonymity

Our proposed scheme can preserve the identity anonymity since *ID*_*i*_ cannot be derived from *M*_1_ without the knowledge of *K*; furthermore, *K* cannot be derived from *Y*_*i*_ without the random number *y*_*i*_ and the pre-shared secret key *PSK*. Also, owing to the one-way hash function, *ID*_*i*_ cannot be derived from *M*_5_. Our proposed scheme therefore provides user anonymity.

#### Resisting outsider attack

Suppose that an adversary A extracts all of the information $\left\{V_{A},W_{A},X_{A},Y_{A}\right\}$ from a smart card by using side channel attack; however, he/she cannot obtain any of the secret information of *S*_*j*_. A can compute $h\left(y_{A}∥PSK\right)=W_{A}⊕ID_{A}$, but the value $y_{A}$ is a random number that is unique to the user that is selected by *RC* and *PSK* is the pre-shared secret key between the *RC* and *S*_*j*_; therefore, A does not know and our proposed scheme can resist an outsider attack.

#### Resisting impersonation attack

Suppose that an adversary A intercepts all of message {*Y*_*i*_,*Z*_*i*_,*M*_1_,*M*_2_,*M*_3_,*M*_4_,*M*_5_,*M*_6_,*T*_1_,*T*_2_,*T*_3_} that are transmitted over a public channel between *U*_*i*_ and *S*_*j*_; however, A cannot generate the legal login request message {*Y*_*i*_,*Z*_*i*_,*M*_1_,*M*_2_,*M*_3_,*T*_1_}, where *Y*_*i*_ = *y*_*i*_ ⊕ *h*(*PSK*), *Z*_*i*_ = *h*(*X*_*i*_ ∥ *n*_1_ ∥ *PWD*_*i*_ ∥ *T*_1_), *M*_1_ = *K* ⊕ *ID*_*i*_, *M*_2_ = *n*_1_ ⊕ *K* and *M*_3_ = *PWD*_*i*_ ⊕ *K*, because the value *y*_*i*_ is a random number that is unique to the user that is selected by the *RC* and *n*_1_ is a random number that is generated by *U*_*i*_; furthermore, A cannot generate the login response message {*M*_4_,*M*_5_,*T*_2_} without the random number *n*_2_. Our proposed scheme can therefore resist an impersonation attack.

#### Session key agreement

Suppose that an adversary A intercepts all of the message {*Y*_*i*_,*Z*_*i*_,*M*_1_,*M*_2_,*M*_3_,*M*_4_,*M*_5_,*M*_6_,*T*_1_,*T*_2_,*T*_3_} that are transmitted over a public channel between *U*_*i*_ and *S*_*j*_, steals the smart card of *U*_*i*_, and then extracts the all information {*V*_*i*_,*W*_*i*_,*X*_*i*_,*Y*_*i*_,*h*(⋅),*H*(⋅)}; however, A cannot compute the session key *SK*_*ij*_ = *h*(*n*_1_ ∥ *n*_2_ ∥ *K* ∥ *X*_*i*_). To compute *K* from *W*_*i*_, the *U*_*i*_’s identity *ID*_*i*_ is needed. To retrieve *ID*_*i*_ from *V*_*i*_, A needs to know *PW*_*i*_ and *H*(*BIO*_*i*_). Since only *U*_*i*_ can imprint the biometrics *BIO*_*i*_ at the sensor, an adversary A cannot attain the *U*_*i*_’s identity *ID*_*i*_ and *PW*_*i*_. Our proposed scheme can therefore provide session key security.

### Formal security analysis

In this subsection, we demonstrate the formal security analysis of our proposed scheme and show that it is secure. First, we define the following hash function [29].

**Definition 1.** A secure one-way hash function *h*: {0, 1}* → {0, 1}^*n*^, which takes an input as an arbitrary length binary string *x* ∈ {0, 1}* and outputs a binary string *h*(*x*) ∈ {0, 1}^*n*^, satisfies the following requirements: *a*. Given *y* ∈ *Y*, it is computationally infeasible to find an *x* ∈ *X* such that *y* = *h*(*x*):*b*. Given *x* ∈ *X*, it is computationally infeasible to find another *x*′ ≠ *x* ∈ *X*, such that *h*(*x*′) = *h*(*x*):*c*. It is computationally infeasible to find a pair (*x*′,*x*) ∈ *X*′ × *X*, with *x*′ ≠ *x*, such that *h*(*x*′) = *h*(*x*).

**Theorem 1.** Under the assumption that the one-way hash function *h*(⋅) closely behaves like an oracle, then our proposed scheme is provably secure against an adversary A for the protection of a user’s personal information including the identity *ID*_*i*_, password *PW*_*i*_ and biometrics *BIO*_*i*_, a server’s secret number *x* that is selected by the *RC* and a pre-shared secret key *PSK* that is between the *RC* and *S*_*j*_.

**Proof.** The formal security proof of our proposed scheme is similar to those in [23, 29, 30]. Using the following oracle to construct A who will have the ability to derive the user *U*_*i*_’s identity *ID*_*i*_, password *PW*_*i*_, biometrics *BIO*_*i*_, the server’s secret number *x* that is selected by the *RC*, and a pre-shared secret key *PSK* between the *RC* and *S*_*j*_.

Reveal: This random oracle will unconditionally output the input *x* from the given hash result *y* = *h*(*x*).

Now, A runs the experimental algorithm that is shown in Table 4, $EXP_{HASH,A}^{JKMSE}$ for our proposed scheme JKMSE.

**Table 4:** Algorithm $EXP_{HASH,A}^{JKMSE}$.

1. Eavesdrop login request message {*Y*_*i*_,*Z*_*i*_,*M*_1_,*M*_2_,*M*_3_,*T*_1_}
2. Call the Reveal oracle. Let $\left(n_{1}^{\prime },X_{i}^{\prime },PWD_{i}^{\prime }\right)\leftarrow Reveal\left(Z_{i}\right)$
3. Eavesdrop login response message {*M*_4_,*M*_5_,*T*_2_}
4. Call the Reveal oracle. Let $\left(ID_{i}^{\prime },n_{1}^{\prime \prime },n_{2}^{\prime },K^{\prime },T_{2}\right)\leftarrow Reveal\left(M_{5}\right)$
5. **if $\left(n_{1}^{\prime }=n_{1}^{\prime \prime }\right)$ then**
6. Call the Reveal oracle. Let $\left(PW_{i}^{\prime },BIO^{\prime }\right)\leftarrow Reveal\left(PWD_{i}^{\prime }\right)$
7. Call the Reveal oracle. Let $\left(ID_{i}^{\prime },x^{\prime }\right)\leftarrow Reveal\left(X_{i}^{\prime }\right)$
8. Compute $K^{\prime \prime }=M_{2}⊕n_{1}^{\prime }$
9. **if** (*K*′ = *K*′′) **then**
10. Call the Reveal oracle. Let $(h^{\prime }(y_{i}^{\prime }||PSK^{\prime }),SID_{j})\leftarrow Reveal\left(K\right)$
11. Compute $n_{2}^{\prime \prime }=M_{4}⊕h\left(n_{1}^{\prime }∥X_{i}∥PWD_{i}^{\prime }\right)$
12. **if** $\left(n_{2}^{\prime }=n_{2}^{\prime \prime }\right)$ **then**
13. Call the Reveal oracle. Let $(y_{i}^{\prime }||PSK^{\prime })\leftarrow Reveal(h^{\prime }(y_{i}\left||PSK)\right)$
14. Accept $ID_{i}^{\prime }$, $PW_{i}^{\prime }$, $BIO_{i}^{\prime }$, $y_{i}^{\prime }$ as the correct *ID*_*i*_, *PW*_*i*_,*BIO*_*i*_ and *y*_*i*_ of *U*_*i*_,*x*′ and *PSK*′ as the correct secret number of *S*_*j*_ and pre-shared secret key between *RC* and *S*_*j*_
15. **return** 1
16. **else**
17. **return** 0
18. **end if**
19. **else**
20. **return** 0
21. **end if**
22. **else**
23. **return** 0
24. **end if**

If the success probability of $EXP_{HASH,A}^{JKMSE}$ is defined as $Success_{HASH,A}^{JKMSE}=\left|Pr\left[EXP_{HASH,A}^{JKMSE}=1\right]-1\right|$, the advantage function for this experiment then becomes $Adv_{HASH,A}^{JKMSE}\left(t,q_{R}\right)=max_{A}Success_{HASH,A}^{JKMSE}$, where the maximum is taken over all of A with the execution time *t* and the number of queries *q*_*R*_ that are made to the Reveal oracle. Consider the experiment that is shown in Table 4 for A. If A has the ability to solve the hash function problem that is provided in Definition 1, then he/she can directly derive *U*_*i*_’s identity *ID*_*i*_, password *PW*_*i*_, biometrics *BIO*_*i*_, the server’s secret number *x* that is selected by the *RC* and the pre-shared secret key *PSK* that is between the *RC* and *S*_*j*_. In this case, A will discover the complete connections between *U*_*i*_ and *S*_*j*_; however, it is a computationally infeasible problem to invert the input from a given hash value, i.e., $Adv_{HASH,A}^{JKMSE}\left(t\right)\leq \epsilon$, ∀*ϵ* > 0. Then, we have $Adv_{HASH,A}^{JKMSE}\left(t,q_{R}\right)\leq \epsilon$, since $Adv_{HASH,A}^{JKMSE}\left(t,q_{R}\right)$ depends on $Adv_{HASH,A}^{JKMSE}\left(t\right)$. As a result, there is no way for A to discover the complete connections between *U*_*i*_ and *S*_*j*_, and, by deriving (*ID*_*i*_,*PW*_*i*_,*BIO*_*i*_,*y*_*i*_,*x*,*PSK*), our proposed scheme is provably secure against an adversary.

## Functional and performance analysis

In this section, we evaluate the functionality the computational costs comparisons between our proposed scheme and the other related schemes [18–23].

### Functional analysis

Table 5 lists the functionality comparisons of our proposed scheme with the other related schemes. The table shows that the proposed scheme achieves all of the security and functionality requirements and is more secure than the other related schemes.

**Table 5:** Functionality comparison.

	Ours	[23]	[22]	[21]	[20]	[19]	[18]
Provide mutual authentication	Yes	Yes	Yes	No	Yes	Yes	Yes
User anonymity	Yes	No	Yes	Yes	Yes	Yes	Yes
Resist insider attack	Yes	Yes	Yes	Yes	Yes	No	Yes
Resist off-line guessing attack	Yes	Yes	Yes	Yes	Yes	No	Yes
Resist stolen smart card attack	Yes	No	Yes	No	-	Yes	Yes
Resist replay attack	Yes	Yes	No	No	No	No	No
Resist verifier attack	Yes	Yes	Yes	Yes	-	No	Yes
Session key agreement	Yes	No	Yes	Yes	Yes	No	Yes
Efficient password change phase	Yes	Yes	No	No	Yes	No	No

### Performance anaylsis

For the performance comparison, the definitions of *T*_*E*_ and *T*_*H*_ are the performance times of a symmetric encryption/decryption operation and a hash function, respectively. Recently, Xue and Hong [31] estimated the running time of different cryptographic operations whereby *T*_*E*_ is nearly 0.45 ms on average, and *T*_*H*_ is below 0.2 ms on average in the environment (CPU: 3.2 GHz, RAM: 3.0 G). Table 6 shows a comparison of the computational costs of the proposed scheme with the other related schemes. In the performance comparison, the proposed scheme requires a greater amount of computation to accomplish mutual authentication and the key agreement than Chuang et al.’s scheme as the proposed scheme performs four further hash operations; however, these operations consume a very small amount of time.

**Table 6:** Computational costs comparison.

Schemes	Registration	Login	Authentication	Total	Time(ms)
Li et al. [18]	6*T*_*H*_	6*T*_*H*_	12*T*_*H*_	24*T*_*H*_	4.8
Xue et al. [19]	7*T*_*H*_	6*T*_*H*_	17*T*_*H*_	30*T*_*H*_	6.0
Lu et al. [20]	6*T*_*H*_	5*T*_*H*_	13*T*_*H*_	24*T*_*H*_	4.8
Chuang et al. [21]	3*T*_*H*_	4*T*_*H*_	13*T*_*H*_	20*T*_*H*_	4.0
Mishra et al. [22]	7*T*_*H*_	4*T*_*H*_	11*T*_*H*_	22*T*_*H*_	4.4
Lu et al. [23]]	5*T*_*H*_	6*T*_*H*_	12*T*_*H*_	23*T*_*H*_	4.6
Our proposed	5*T*_*H*_	5*T*_*H*_	13*T*_*H*_	23*T*_*H*_	4.6

*T*_*H*_: hash function evaluation

## Conclusion

In this paper, we analyzed the security weaknesses of a biometrics-based authentication scheme for multi-server environments by Lu et al. Lu et al. claimed that their authentication scheme is secure and provides user anonymity; however, we found that Lu et al.’s scheme is still insecure against outsider attacks and impersonation attacks. To resolve these security vulnerabilities, we proposed an improved protocol for an authentication scheme that retains the merits of Lu et al.’s scheme and also achieves a comprehensive security. The security analysis of this paper explains that the proposed scheme rectifies the weaknesses of Lu et al.’s scheme.
